# PRKCA Overexpression Is Frequent in Young Oral Tongue Squamous Cell Carcinoma Patients and Is Associated with Poor Prognosis

**DOI:** 10.3390/cancers13092082

**Published:** 2021-04-25

**Authors:** Thomas Parzefall, Julia Schnoell, Laura Monschein, Elisabeth Foki, David Tianxiang Liu, Alexandra Frohne, Stefan Grasl, Johannes Pammer, Trevor Lucas, Lorenz Kadletz, Markus Brunner

**Affiliations:** 1 Department of Otorhinolaryngology, Head and Neck Surgery, Medical University of Vienna, 1090 Vienna, Austria; thomas.parzefall@meduniwien.ac.at (T.P.); julia.schnoell@meduniwien.ac.at (J.S.); elisabeth.foki@meduniwien.ac.at (E.F.); david.liu@meduniwien.ac.at (D.T.L.); stefan.grasl@meduniwien.ac.at (S.G.); markus.brunner@meduniwien.ac.at (M.B.); 2 Institute of Pathology, Medical University of Vienna, 1090 Vienna, Austria; laura.monschein@chello.at (L.M.); johannes.pammer@meduniwien.ac.at (J.P.); 3 Department for Cell and Developmental Biology, Center for Anatomy and Cell Biology, Medical University of Vienna, 1090 Vienna, Austria; alexandra.frohne@meduniwien.ac.at (A.F.); trevor.lucas@meduniwien.ac.at (T.L.)

**Keywords:** oral tongue squamous cell carcinoma, early onset, high risk, prognostic marker, protein kinase c alpha, PRKCA

## Abstract

**Simple Summary:**

Over the last decades, the incidence of tongue cancer has risen among young patients who often show an aggressive course of disease. At the same time, exposure to the major risk factors, alcohol and tobacco, has decreased, indicating that a novel risk factor may be involved. In this study, we found that high expression of protein kinase C alpha (PRKCA) is frequent among young patients without alcohol and smoking history and associated with a poor prognosis. Our results suggest that PRKCA levels may serve as a molecular marker of an emerging high-risk subgroup of young tongue cancer patients. Elucidation of the underlying mechanisms may clarify whether PRKCA expression itself promotes disease progression and which genetic or environmental factors trigger its upregulation.

**Abstract:**

Oral tongue squamous cell carcinomas (OTSCCs) have an increasing incidence in young patients, and many have an aggressive course of disease. The objective of this study was to identify candidate prognostic protein markers associated with early-onset OTSCC. We performed an exploratory screening for differential protein expression in younger (≤45 years) versus older (>45 years) OTSCC patients in The Cancer Genome Atlas (TCGA) cohort (*n* = 97). Expression of candidate markers was then validated in an independent Austrian OTSCC patient group (*n* = 34) by immunohistochemistry. Kaplan–Meier survival estimates were computed, and genomic and mRNA enrichment in silico analyses were performed. Overexpression of protein kinase C alpha (PRKCA) was significantly more frequent among young patients of both the TCGA (*p* = 0.0001) and the Austrian cohort (*p* = 0.02), associated with a negative anamnesis for alcohol consumption (*p* = 0.009) and tobacco smoking (*p* = 0.02) and poorer overall survival (univariate *p* = 0.02, multivariate *p*< 0.01). Within the young subgroup, both overall and disease-free survival were significantly decreased in patients with PRKCA overexpression (both *p* < 0.001). TCGA mRNA enrichment analysis revealed 332 mRNAs with significant differential expression in PRKCA-upregulated versus PRKCA-downregulated OTSCC (all FDR ≤ 0.01). Our findings suggest that PRKCA overexpression may be a hallmark of a novel molecular subtype of early-onset alcohol- and tobacco-negative high-risk OTSCC. Further analysis of the molecular PRKCA interactome may decipher the underlying mechanisms of carcinogenesis and clinicopathological behavior of PRKCA-overexpressing OTSCC.

## 1. Introduction

Head and neck squamous cell carcinomas (HNSCCs) are the sixth most common cancer, with a worldwide annual incidence of more than 800,000 cases [1]. Major risk factors include chronic alcohol and tobacco consumption, promoting mutagenesis, chromosomal instability, progressive epithelial dysplasia and carcinoma formation [2,3,4], and, specific to oropharyngeal carcinoma, infection with human papilloma virus (HPV). HNSCCs typically have a peak incidence around the sixth to seventh decade of life [5,6].

While the incidence of tobacco- and alcohol-related HNSCC has decreased over the past decades in many Western countries, HPV-associated carcinomas of the oropharynx have followed an opposite trend [7] and are typically diagnosed around the age of 50.

A notable worldwide increase is also observed in the incidence of oral tongue squamous cell carcinoma (OTSCC) among young individuals [8,9,10]. Since many of these cases appear to be linked to none of the established risk factors, additional pathomechanisms may be at play in this particular patient subgroup [6,11,12,13,14]. While data on the prognosis and biological behavior of OTSCC in different age groups remain ambiguous, younger patients have been reported to have higher rates of regional and distant metastasis and a highly aggressive course of disease in recurrent cases [15,16,17]. The identification of molecular markers in early-onset OTSCC may help to identify high-risk patients, clarify novel mechanisms of disease, characterize the clinicopathologic tumor behavior and may eventually lead to improved prognoses and treatment strategies.

In recent years, multiple comparative studies have investigated genomic and transcriptomic properties of OTSCC tumors between younger and older patients. Thus far, attempts to link specific genetic variants to either age group have remained unsuccessful [18,19]. However, one study reported an association of a high DNA copy number variant (CNV) burden with reduced overall survival in the young patient subgroup [20]. Additionally, OTSCC tumors from younger and older patients were found to differ in mRNA expression patterns of the immunomodulatory markers LAG3 and HAVCR2 [21], and young patients, as well as non-smokers, were reported to carry fewer p53 mutations [22,23]. On the mRNA and protein level, altered cell cycle regulation due to p53 overexpression, angiogenesis promotion via upregulation of HIF1a, deregulation of gene expression via Sox2 overexpression and epithelial-mesenchymal transition (EMT) through upregulation of Vimentin and downregulation of Cadherin E have also been evaluated as molecular negative prognostic markers in OTSCC samples using immunohistochemistry or targeted tissue micro-arrays [22,24,25,26,27,28,29,30,31]. However, the results vary, and studies focusing on young OTSCC patients are scarce.

In contrast to targeted investigations of pre-selected proteins or protein sets, large-scale proteomics approaches using mass spectrometry allow a broad and unbiased examination of tumor protein expression and detection of novel protein markers. The Cancer Genome Atlas (TCGA) database (https://www.cancer.gov/tcga; last accessed on 1 March 2021) gives access to a unique abundance of comprehensive clinical and molecular data from cancer patients, tumor and control tissues, including for OTSCC.

The objective of the present study was to identify candidate molecular markers associated with early-onset OTSCC with a poor prognosis. For this purpose, we used TCGA data to screen for protein markers differentially expressed in young OTSCC patients compared to older patients. The data gained from this initial exploratory TCGA screening served as a basis for the further targeted evaluation of protein expression in a local Austrian OTSCC patient group and upstream genomic and transcriptomic in silico analyses.

## 2. Patients and Methods

### 2.1. Study Design and Setting

The study was designed as a two-step retrospective observational cohort study. In the first step, protein markers associated with young age (≤45 years) in patients with OTSCC were explored within the TCGA data sets. In the second step, identified candidate protein markers were experimentally validated in an independent OTSCC cohort treated at an Austrian tertiary referral center. The main outcome measure was the association of any candidate protein marker with an onset age of ≤45 years. The secondary outcome measures were the association of any identified marker with alcohol and tobacco smoking anamnesis and the overall (OS) and disease-free survival (DFS).

### 2.2. TCGA Data Retrieval and Selection

TCGA HNSCC datasets were retrieved via the cBio Cancer Genomics Portal [32]. Samples from all OTSCC patients with proteomic (RPPA and z-scores), mRNA, DNA and clinical data available were selected for analysis. Data were extracted from three distinct data sets within TCGA (HNSCC, TCGA, Provisional; HNSCC, TCGA PanCancer Atlas; and HNSCC, TCGA, Nature 2015) and duplicate samples across datasets were excluded from the analysis. The data were initially sorted by the patient age at disease onset and divided into two groups of young (≤45 years) and older (>45 years) patients. The age threshold at 45 years was adopted from previous studies related to early-onset OTSCC [9,15,21].

### 2.3. Austrian Patient Sample

Tissue samples from newly diagnosed, previously untreated patients with OTSCC, obtained during primary surgical resection or diagnostic panendoscopy at the Department of Otorhinolaryngology, Head and Neck Surgery, Medical University of Vienna, between 1999 and 2016, were retrieved from the local tissue archive. All samples with a sufficient tissue volume to obtain three slides of 4 µm (1× primary antibody immunostaining, 1× isotype control, 1× hematoxylin/eosin only) were included in the analysis. Patients with a previous history of malignant disease or any synchronous malignancy were excluded. Additionally, the clinical parameters age, sex, AJCC tumor staging (seventh edition), treatment modalities, history of alcohol and/or tobacco consumption, and clinical outcome were extracted from the patient records. The study was conducted in concordance with the WMA Declaration of Helsinki and was approved by the Ethics Committee of the Medical University of Vienna (approval no. 1262/2019).

### 2.4. Smoking and Alcohol Consumption Status in the Patient Samples

For the statistical analysis, tobacco smoking and alcohol consumption statuses were coded in a binary fashion. Patients with a self-reported lifelong cumulative smoking history of less than 100 cigarettes were coded as non-smokers, in concordance with the NHI National Cancer Institute definitions (https://cdebrowser.nci.nih.gov/cdebrowserClient/cdeBrowser.html#/search?publicId=2181650&version=1.0 (accessed on 1 March 2021)). Current and reformed smokers with a cumulative dose exceeding 100 cigarettes were considered smokers. Regarding alcohol status, subjects with a consumption of less or equal to two alcoholic beverages/week (social- and never-drinkers) were considered alcohol-negative. Three or more alcoholic beverages per week were considered to indicate positive alcohol anamnesis. Patients with an unknown tobacco and/or alcohol status were not included in the analysis.

### 2.5. Immunohistochemistry

Immunohistochemical (IHC) staining of archived formalin-fixed and paraffin-embedded tissue sections was performed using a Lab Vision Ultra kit (Thermo Fisher Scientific, Waltham, MA, USA) according to the manufacturer’s protocol. Initially, the ideal antibody dilutions (1:800, Rabbit MAB Anti-PRKCA, AB no. ab32376, Abcam, Cambridge, UK, and 1:400, Mouse MAB Anti-ANXA1, AB no. EH17a, Developmental Studies Hybridoma Bank, University of Iowa, IA, USA) and retrieval buffers were assessed using human cerebral and esophageal samples, respectively. These samples were also used as positive controls. Tissue samples were dewaxed and rehydrated using xylol, ethanol and water. Endogenous peroxidase activity was blocked in 3% H_2_O_2_ for 15 min. Antigen retrieval was performed in a microwave (600 W) using citrate buffer (pH 6.0). Subsequently, Ultra V Block was applied for five minutes. Then, the tissue samples were incubated with the primary antibodies at room temperature for one hour. Next, the primary antibody enhancer and horseradish peroxidase enhancer were applied for 10 and 15 min, respectively. Antibody staining was visualized using the UltraVision Plus Detection System DAB Plus Substrate System (Thermo Fisher Scientific, Waltham, MA, USA), and samples were counterstained using hematoxylin Gill II (Merck, Darmstadt, Germany). For negative controls, the primary antibody was replaced with rabbit immunoglobulin G isotype control (Abcam, Cambridge, UK).

### 2.6. Protein Expression Quantification

In the TCGA cohort, protein expression fold changes with a z-score of ≥+ 1.96 or ≤−1.96 (*p* ≤ 0.05) were considered as overexpression and underexpression, respectively. In the Viennese OTSCC samples, semiquantitative analysis of immunohistochemically stained tissue sections was performed in a consensus manner by two experienced pathologists (L.M., J.P.) who were blinded to the clinical patient data. Both the fraction of positively stained carcinoma cells and the expression intensity were measured to classify protein expression levels. Samples were graded according to the fraction of positive cells into 0 (<5% positive cells), 1 (5–33% positive cells), 2 (>33–66% positive cells), 3 (>66% positive cells) and according to the staining intensity into 0 (none), 1 (weak), 2 (moderate), 3 (strong). Positive cell fraction and intensity scores were summed up to give final IHC scores of 0 (min.) to 6 (max.). Based on an a priori definition, any IHC score above the average value on the scale (≥4) was considered overexpression.

### 2.7. Statistical Analysis

Two-tailed Fisher’s exact test was used to determine the statistical association of target protein differential expression and young age (statistical cut-off: *p* ≤ 0.01) as well as alcohol/tobacco consumption (positive versus negative consumption history) in both the TCGA and the Vienna cohort. For survival analysis, Kaplan–Meier estimates were computed. The median survival time was calculated as the shortest survival time for which the survivor function was ≤ 50%. Accordingly, if the survivor function remained >50% the median survival time was termed as undefined. Intergroup differences were assessed with log-rank tests. Multivariate survival analysis on the combined cohort (TCGA and Vienna) was performed using a Cox-regression model including patient age, T-classification, N-classification and PRKCA protein expression status. All statistical calculations were carried out with Stata (StataCorp, College Station, TX, USA) and Prism GraphPad (GraphPad Software, San Diego, CA, USA). Comparative genomic analysis between PRKCA-high and PRKCA-low TCGA samples was conducted with the integrated sample comparison function of the cBio Cancer Genomics Portal [32] with the Student’s *t*-test. Correction for multiple testing was performed with the Benjamini–Hochberg procedure with an accepted false detection rate of 5% (*p*-value ≤ 0.05).

### 2.8. Differential mRNA Expression and Gene Ontology Enrichment Analysis

Datasets containing RNA-Seq (RSEM) results were retrieved through the National Cancer Institute (NCI) Genomic Data Commons (GDC) Application Programming Interface (API) with the R/Bioconductor package TCGAbiolinks [33,34]. The data were normalized with TCGAanalyze_Normalization (using EDASeq, [35] and filtered with TCGAanalyze_Filtering (quantile filter 0.25). Differential mRNA expression was tested between PRKCA-high and PRKCA-low (protein expression) tumor samples using the edgeR functions (DGEL, estimateCommonDisp, exactTest, topTags) integrated into TCGAanalyze_DEA with an FDR cut-off at ≤0.01. A gene ontology (GO) enrichment analysis was performed using the geneontology.org/ web interface (accessed on 11 April 2021) with Fisher’s exact test and an FDR cut-off at 0.05. As a reference, a file containing identifiers of all genes included in the differential expression analysis was uploaded.

## 3. Results

### 3.1. Patient Characteristics

Within the TCGA HNSCC data, a total of 98 OTSCC patient records contained information about both clinical parameters and proteomics data and were included in the analysis (Supplemental Table S1). The patient characteristics of the TCGA cohort and the Viennese cohort are summarized in Table 1. The TCGA cohort consisted of 63 male and 35 female patients ranging from 19 to 87 years (mean: 57.5; SD: 13.6); 15 patients were 45 years or younger, and 82 patients were older than 45 years. The age was unknown in one TCGA sample (TCGA-CQ-A4CA-01), which was therefore excluded from any age-related statistical calculation. The Viennese group consisted of 34 (20 male, 14 female) patients with an age range of 20 to 75 years (mean: 49.5; SD: 15.9) at the time of first diagnosis. There were 14 patients 45 years or younger, and 20 patients were older than 45 years.

### 3.2. PRKCA Is Frequently Overexpressed in Young OTSCC Patients

**Figure 1:**
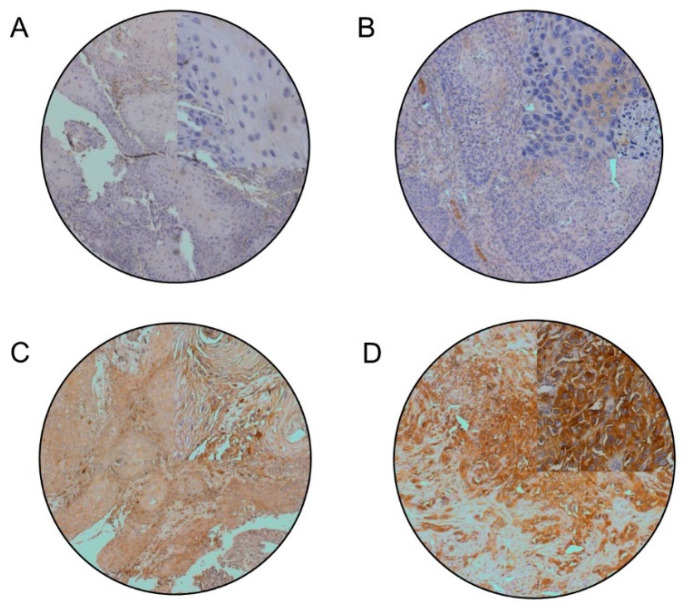
Representative IHC images of OTSCC tissue stained for PRKCA. Examples of OTSCC with (**A**) no, (**B**) weak focal, (**C**) moderate and (**D**) strong PRKCA immunoreactivity (10×). Areas in the upper right quadrants were enlarged to 40×.

In the initial TCGA screening, two proteins, Protein kinase C alpha (PRKCA) and Annexin 1 (ANXA1), met the criteria for further experimental validation (two-tailed Fisher’s exact test, *p* ≤ 0.01). Subsequently, ANXA1 overexpression was found not statistically overrepresented in younger compared to older patients in the Viennese validation samples (*p* = 1.0). However, PRKCA overexpression was found to be significantly more frequent in young (≤45 years) compared to older (>45 years) patients in both the TCGA cohort (*n* = 97; *p* = 0.0001) and the Vienna validation study group (*n* = 34; *p* = 0.02) (Figure 1). In the TCGA cohort, six out of 15 patients (40%) aged ≤45 years had a significant PRKCA overexpression with a z-score above +1.96, whereas among the patients >45 years, two out of 82 patients (2.4%) showed PRKCA overexpression. The median PRKCA IHC score in the Vienna patients ≤45 years was 0.5 (range 0–6). In the group >45 years, the median total IHC score was 0 (range 0–3). Four out of 14 young patients (28.6%), as opposed to none of the patients > 45 years (0%), had a total IHC score of ≥4 (PRKCA upregulated).

### 3.3. PRKCA Overexpression Is Associated with Adverse Clinical Outcome

**Figure 2:**
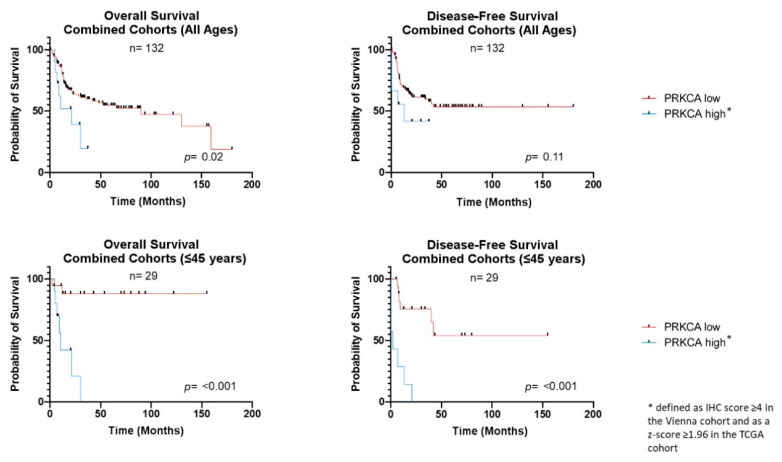
Kaplan–Meier survival curves in correlation to PRKCA expression levels. Univariate survival analysis shows significantly reduced OS of OTSCC patients with high PRKCA expression (*p* = 0.02). In the subgroup of patients aged 45 years or younger, high PRKCA expression compromises both OS and DFS (each *p* < 0.001).

To investigate whether differential expression of the candidate proteins PRKCA and ANXA1 have an influence on the clinical outcome, we calculated the Kaplan–Meier survival functions in the TCGA and Viennese cohorts. ANXA1 expression did not show an association with either OS or DFS in the total study population or within any subgroup (Supplemental Figure S1) and was therefore not considered further as a candidate prognostic marker. However, PRKCA overexpression significantly correlated with adverse clinical outcomes (Figure 2, Supplemental Figure S2). In the Vienna cohort, reduced OS and DFS and tumor recurrence at the last follow-up were significantly associated with PRKCA upregulation in the total cohort and the young patient fraction (all univariate *p* ≤ 0.002). Similarly, in the TCGA cohort, DFS was significantly reduced in young OTSCC patients overexpressing PRKCA (univariate *p* = 0.02). Combining both groups resulted in a significant association of PRKCA overexpression with poor DFS (univariate *p* < 0.0001) and OS (univariate *p* < 0.001) in the young patients. Additionally, we performed univariate survival analysis for patient age, AJCC T-classification and N-classification in the combined cohort, which showed a significant association of T-classification and N-classification (both univariate *p* < 0.01) with OS. No significant additional association was found with DFS. In the multivariate analysis, T-classification (*p* < 0.01), N-classification (*p* = 0.05) and PRKCA overexpression (*p* < 0.01) remained significantly associated with OS. None of the analyzed parameters were associated with DFS after multivariate testing (Table 2). A detailed list of all subgroup survival times and calculated univariate and multivariate hazard ratios in regard to PRKCA expression is displayed in Table 3.

### 3.4. PRKCA Overexpression Is Frequent in Alcohol and Tobacco Negative OTSCC

We then calculated the statistical associations of PRKCA overexpression with alcohol and tobacco consumption behavior of the patients. We found an association of PRKCA upregulation with a negative history of alcohol and tobacco consumption in both the Viennese and the TCGA cohort. In the TCGA cohort, this association was statistically significant for alcohol consumption (*p* = 0.01, two-tailed). When combining the data from both the Vienna and the TCGA cohorts, the correlation between both alcohol consumption (*p* = 0.009, two-tailed) and tobacco smoking (*p* = 0.02, two-tailed) became statistically significant.

### 3.5. Messenger RNA Expression Profiles Differ Significantly between PRKCA Positive and PRKCA Negative OTSCC

**Figure 3:**
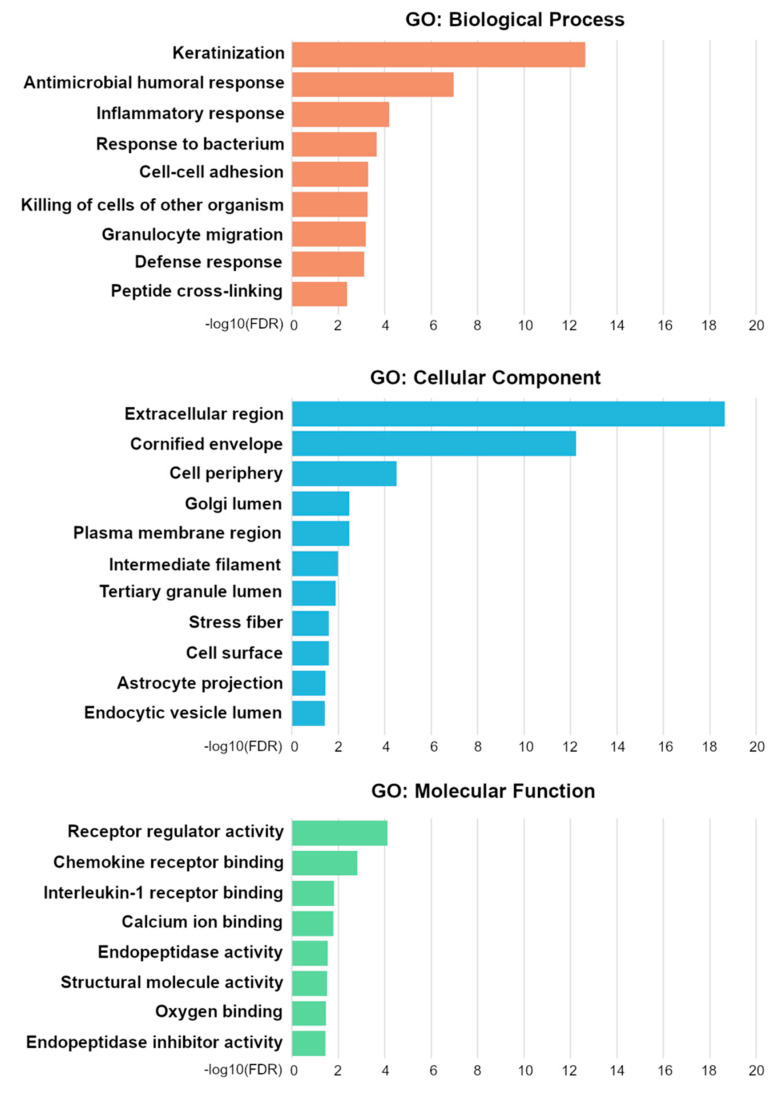
Gene ontology (GO) terms from enrichment analysis of differentially expressed genes. For hierarchically related terms, only the one with the lowest FDR is included. FDR, false detection rate.

To identify potential upstream molecular alterations associated with PRKCA protein overexpression, we compared genomic and transcriptomic data of PRKCA-high and PRKCA-low TCGA samples. Curated DNA sequence and CNV data were available for all except one OTSCC sample (TCGA-CQ-6221) in the HNSCC TCGA PanCancer Atlas data (*n* = 97). Comparative genomic analysis between PRKCA-high (*n* = 8) and -low (*n* = 89) samples within the TCGA records showed no significant differences in the total mutation count, specific DNA sequence variants, or CNV patterns (Supplementary Table S2, Supplementary Figure S3). The mRNA enrichment analysis was performed with all PRKCA-low and seven out of eight PRKCA-high samples since RNA-Seq data for TCGA-CN-A640 were not available. A total of 332 protein-coding genes (Supplementary Table S3) were found to be differentially expressed (FDR ≤ 0.01). GO analysis identified significant (FDR ≤ 0.05) enrichment with 98 terms in the ontology classes GO: Biological Process (52 hits), Cellular Compartment (27 hits), and GO: Molecular Function (19 hits). Due to the hierarchical organization of GO categories, genes associated with related terms were partly redundant. Figure 3 summarizes the GO results and, for each group of related terms, only contains the hierarchical level with the lowest FDR. The full results are shown in Supplementary Table S4.

## 4. Discussion

Over the past decades, a decline in the use of tobacco and alcohol in most Western countries has been accompanied by a decreasing incidence of most HNSCCs [7,8,9,10]. Running counter to this trend, a concurrent rise in cases of early-onset OTSCC has been observed. Since OTSCC were shown not to be appreciably linked to HPV, the cause for this increase is still obscure [12,13,14]. A set of clinical differences, including age, severity, and exposure to established risk factors, have prompted the notion that early-onset and late-onset OTSCC may represent distinct disease subtypes. A higher CNV burden and attenuated anti-tumor immune activity, reflected by lower cytolytic activity scores, fewer neoantigens, and mRNA-level downregulation of immunomodulators such as HAVCR2 and LAG3, have been linked to early-onset OTSCC [20,21]. So far, however, reliable biomarkers are lacking, preventing molecular subtype characterization, development of tailored therapies, and reliable prognoses. In the present study, PRKCA was identified as a protein marker that is significantly overexpressed in a fraction of early-onset OTSCC patients with aggressive courses of disease, indicating that, rather than presenting one group, early-onset OTSCC may comprise at least two disease subtypes, one of which is characterized by high PRKCA expression and poor prognosis.

While previous studies aiming to identify biomarkers have focused on known pre-selected protein markers from other cancer types, we chose an unbiased approach with an initial exploratory screening of TCGA high-throughput proteomics data. Such an approach allows the detection of novel markers but is associated with a higher risk of false positives. Therefore, we validated the results by IHC analysis in a second, independent OTSCC cohort following a rigid protocol, in which the analyzing pathologists were blind to the clinical data of the patients. The combination of the local Viennese and the larger TCGA cohort also allowed to upscale the total sample size and statistical power and to level out sample heterogeneity inherent to retrospective data.

The role of PRKCA as a tumorigenic marker is plausible: Protein kinase C isoforms have been long identified as the intracellular receptors of phorbol esters that promote tumor formation during two-stage chemical-induced carcinogenesis in mouse skin [36,37]. Subsequently, PRKCA has been shown to intersect with the MAPK/ERK and PI3K/AKT pathways, which are frequently active in several cancer types, promoting tumor progression by suppressing apoptosis and inducing proliferation, migration, invasion and angiogenesis [38,39,40,41,42,43,44].

In addition to a more frequent upregulation in younger patients in this study, PRKCA expression correlated with a negative tobacco smoking and alcohol consumption anamnesis and was associated with reduced OS and DFS. While age itself did not have an influence on the prognosis, PRKCA overexpression status showed a highly significant association with poor OS and DFS within the young OTSCC subgroup (Figure 2). Based on these observations, we hypothesized that early-onset OTSCC may be subdivided into high-risk PRKCA-overexpressing and lower-risk PRKCA-negative forms. To further investigate this idea, TCGA mRNA-level expression data were retrieved and tested for differential expression between PRKCA-high and PRKCA-low tumors.

A total of 332 differentially expressed mRNAs were identified, including 138 that were upregulated and 195 that were downregulated in the PRKCA-high group (Supplementary Table S3). A GO enrichment analysis returned multiple terms related to epithelial differentiation and immune functions that have previously been linked to tongue squamous cell carcinoma, including keratinization (GO:0031424), epithelial cell differentiation (GO:0030855), epidermis development (GO:0008544), cell chemotaxis (GO:0060326), and defense response (GO:0006952) [45]. Multiple keratins, constituents of the cornified envelope, and cross-linking proteins had significantly different expression levels between the groups and are also expressed in normal tongue tissue. The dysregulation of keratinization is a common feature of oral carcinoma [46,47] that may be linked to epithelial malignant transformation and a disturbed epithelial barrier function. Inflammatory processes play a central role in the tumor microenvironment, and genes linked to immune functions have emerged from several mRNA and protein enrichment studies between tumor and normal tissue. [48,49,50] Since PRKCA exerts pro-inflammatory effects through stimulation of Th1-cell-derived IFN-alpha and downstream IL1-beta- dependent activation of anti-tumor macrophages, an altered immunological activity in PRKCA-high versus -low tumors appears plausible [51,52,53]. Moreover, PRKCA activates PI3K/AKT signaling which, downstream, promotes EMT, a process crucial for invasion and metastasis that may be linked to the severe course seen in the PRKCA-high group [40,54].

**Figure 4:**
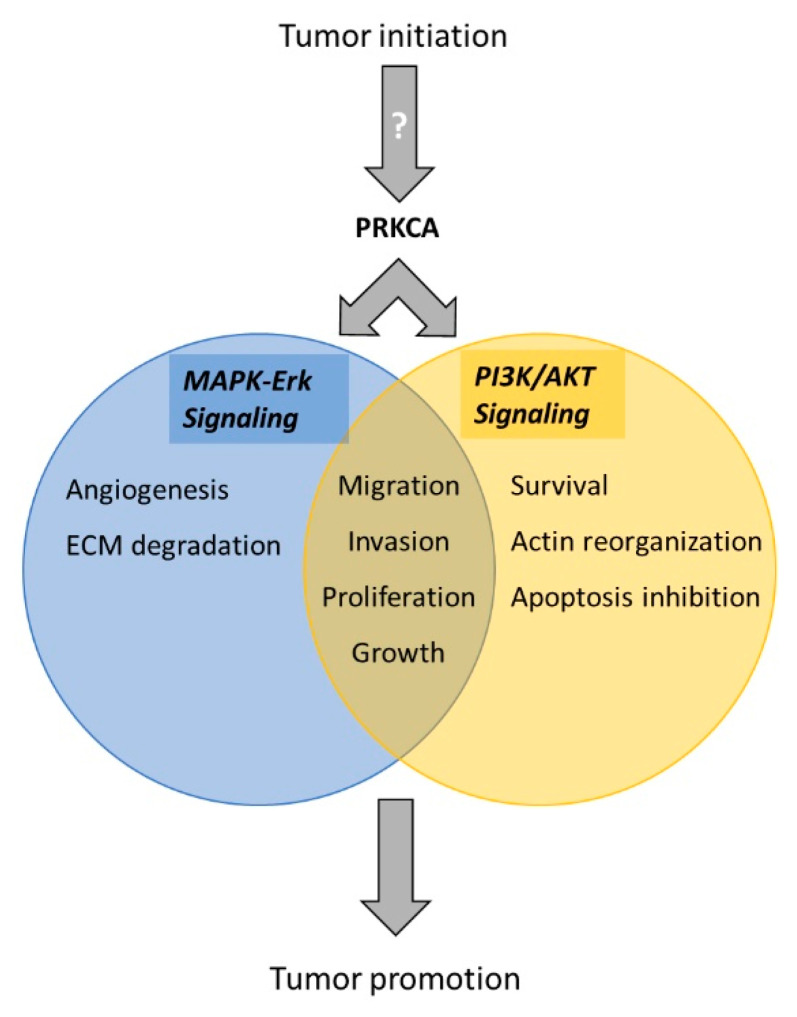
Model of the role of PRKCA in tumorigenesis and progression. Activation of PRKCA is known to elicit downstream tumor-promoting effects via the MAPK/Erk and PI3K/Akt signaling pathways in several cancer types [40,41,42,43,44]. Both pathways are known to promote cell growth, proliferation, migration and invasion. Additionally, MAPK/Erk also promotes angiogenesis and degradation of the extracellular matrix (ECM), while dysregulation of the PI3K/Akt pathway can lead to actin reorganization, improved cellular survival, and inhibition of apoptosis [38,39]. The tumor-initiating mechanism of PRKCA activation and overexpression is unknown.

Based on our findings and the known molecular roles of PRKCA in relevant cancer pathways, we propose a model for PRKCA overexpression as a driver in the tumorigenesis of a subset of OTSCC patients (Figure 4). However, the tumor initiator(s) remain elusive. Given that the DNA mutational landscape does not differ significantly between PRKCA-positive and -negative tumors, specific mutational events or genetic predisposition seem to present unlikely candidates. Rather, the rising incidence of early-onset patients in recent decades could suggest a role of environmental, dietary or lifestyle factors, though alcohol, smoking and HPV do not play a substantial role.

Interestingly, despite a decrease in overall lung carcinoma rates, an increasing incidence has, in recent decades, also been observed for lung adenocarcinoma (LADC) in the USA and China, particularly among younger females, and PRKCA overexpression has also been associated with lower survival rates in LADC [55,56,57]. These findings further support the hypothesis that independent risk factors, which, directly or indirectly, trigger PRKCA upregulation may remain to be identified.

Our results may have therapeutic implications since several compounds have been under clinical investigation to target overexpression of different PRKC isoforms, including PRKCA, in cancer patients. Strategies include inhibition of upstream regulators, small molecule competitive inhibitors or antisense oligonucleotides [58].

## 5. Conclusions

Our results suggest that PRKCA overexpression may define a distinct subtype of early-onset OTSCC with poor prognosis and a yet-unknown mechanism of carcinogenesis. The occurrence of distinct early-onset OTSCC subtypes and varying subtype distributions in study cohorts would offer a possible explanation for controversial reports about survival in young OTSCC patients.

## Figures and Tables

**Table 1:** Clinicopathologic features of the study samples. Abbreviations: class., classification; unkn., unknown; CR, chemoradiation; RT, radiotherapy; EPT, electroporation therapy; Neck diss., neck dissection; PORT, postoperative radiotherapy; SS, salvage surgery (after EPT).

Variable	TCGA (*n* = 98)		Vienna (*n* = 34)		Combined (*n* = 132)	
*Age (years)*	≤45 (*n* = 15)	>45(*n* = 82) ^a^	≤45 (*n* = 14)	>45 (*n* = 20)	≤45 (*n* = 29)	>45 (*n* = 103)
*Sex*						
Male Female	8 (53.3%) 7 (46.7%)	55 (66.3%) 28 (33.7%)	9 (64.3%) 5 (35.7%)	11 (55%) 9 (45%)	17 (58.6%) 12 (41.4%)	66 (64.1%) 37 (35.9%)
*T class.* ^b^						
Tx T1 T2 T3 T4	0 3 (20%) 6 (40%) 3 (20%) 3 (20%)	3 (2.6%) 11 (13.3%) 31 (37.3%) 26 (31.3%) 12 (14.5%)	1 (7.1%) 6 (42.9%) 4 (28.6%) 1 (7.1%) 2 (14.3%)	0 7 (35%) 8 (40%) 2 (10%) 3 (15%)	1 (3.4%) 9 (31.1%) 10 (34.5%) 4 (13.8%) 5 (17.2%)	3 (2.9%) 18 (17.5%) 39 (37.9%) 28 (27.2%) 15 (14.5%)
*N class.*						
Nx N0 N1 N2 N3	0 8 (53.4%) 2 (13.3%) 5 (33.3%) 0	4 (4.8%) 40 (48.2%) 16 19.3%) 23 (27.7%) 0	1 (7.1%) 9 (64.3%) 2 (14.3%) 2 (14.3%) 0	0 14 (70%) 4 (20%) 2 (10%) 0	1 (3.4%) 17 (58.7%) 4 (13.8%) 7 (24.1%) 0	4 (3.9%) 54 (52.4%) 20 (19.4%) 25 (24.3%) 0
*M class.*						
Mx M0 M1	10 (66.7%) 5 (33.3%) 0	58 (69.9%) 25 (30.1%) 0	5 (35.7%) 9 (64.3%) 0	3 (15%) 17 (85%) 0	15 (51.7%) 14 (48.3%) 0	61(59.2%) 42 (40.8%) 0
*Tobacco*						
Yes No Unkn.	10 (66.7%) 5 (33.3%) 0	26 (31.3%) 56 (67.5%) 1 (1.2%)	10(71.5%) 3 (21.4%) 1 (7.1%)	6 (30%) 14 (70%) 0	20 (69%) 8 (27.6%) 1 (3.4%)	32 (31.1%) 70 (68%) 1 (0.9%)
*Alcohol*						
Yes No Unkn.	1 (6.7%) 6 (40%) 8 (53.3%)	30 (36.1%) 5 (6%) 48 (57.9%)	2 (14.3%) 11(78.6%) 1 ((7.1%)	10 (50%) 10 (50%) 0	3 (10.3%) 17 (58.7%) 9 (31%)	40 (38.8%) 15 (14.6%) 48 (46.6%)
*Primary treatment*						
Surgery CR RT EPT	15 (100%) 0 0 0	83 (100%) 0 0 0	11(78.6%) 2 (14.3%) 1 (7.1%) 0	9 (45%) 3 (15%) 2 (10%) 6 (30%)	26 (89.7%) 2 (6.9%) 1 (3.4%) 0	92 (89.3%) 3 (2.9%) 2 (1.9%) 6 (5.8%)
*Neck diss.*						
Yes No	13 (86.7%) 2 (13.3%)	76 (91.6%) 7 (8.4%)	10(71.4%) 4 (28.6%)	10 (50%) 10 (50%)	23 (79.3%) 6 (20.7%)	86 (83.5%) 17 (16.5%)
*Adjuvant treatment*						
PORT CR SS None	n/a n/a n/a n/a	n/a n/a n/a n/a	5 (35.8%) 1 (7.1%) 0 8 (57.1%)	1 (5%) 0 3 (15%) 16 (80%)	n/a n/a n/a n/a	n/a n/a n/a n/a

^a^ Age is unknown in one sample (TCGA-CQ-A4CA), ^b^ according to AJCC seventh edition.

**Table 2:** Overview of unadjusted and adjusted hazard ratios for key clinico-pathological parameters.

**Overall Survival (Combined Cohorts, All Ages)**								
**Analysis**	**Univariate**				**Multivariate**			
**Parameter**	Hazard Ratio	Lower 95%	Upper 95%	*p*-value	Hazard Ratio	Lower 95%	Upper 95%	*p*-value
*Age*	1.01	0.99	1.03	0.19	1.02	0.99	1.04	0.15
*T class*	1.86	1.41	2.44	<0.01	1.80	1.31	2.45	<0.01
*N class*	1.38	1.15	1.64	<0.01	1.24	0.99	1.2	0.05
*PRKCA*	3.62	1.18	11.45	0.02	3.57	1.53	8.56	<0.01
*Smoking*	1.54	0.75	4.45	0.29	1.04	0.95	1.14	0.46
*Sex*	1.08	0.89	1.24	0.45	1.07	0.91	1.15	0.48
**Disease-Free Survival (Combined Cohorts, All Ages)**								
**Analysis**	**Univariate**				**Multivariate**			
**Parameter**	Hazard Ratio	Lower 95%	Upper 95%	*p*-value	Hazard Ratio	Lower 95%	Upper 95%	*p*-value
*Age*	0.98	0.95	1.01	0.58	0.99	0.95	1.02	0.62
*T class*	0.72	0.34	1.49	0.26	0.86	0.40	1.83	0.70
*N class*	0.58	0.23	1.39	0.22	0.61	0.23	1.58	0.31
*PRKCA*	2.82	0.78	10.10	0.11	1.68	0.25	13.23	0.55
*Smoking*	0.98	0.68	5.03	0.55	0.97	0.88	4.66	0.58
*Sex*	1.24	0.66	3.32	0.69	1.13	0.76	3.02	0.64

**Table 3:** Median overall and disease-free survival times and hazard ratios in the patient subgroups as a function of PRKCA overexpression.Abbreviations: PRKCA, protein kinase C alpha; UV, univariate; MV, multivariate; HR, hazard ratio; CI, confidence interval; OS, overall survival; DFS, disease-free survival; TU, tumor; LFU, last follow-up; undef., undefined.

Cohort	Median Survival ^a^ (Months)		UV HR (95% CI)	UV *p*-Value ^c^	MV HR (95% CI)	MV *p*-Value ^d^
	*PRKCA positive* ^b^	*PRKCA negative*				
Vienna All OS	15	130	34.34 (4.15–284.1)	0.001	-	-
Vienna Young OS	15	Undef. ^a^	141.8 (12.24–1642)	<0.001	-	-
Vienna All DFS	0.5	Undef. ^a^	107.7 (10.03–1158)	<0.001	-	-
Vienna Young DFS	0.5	Undef. ^a^	31.15 (3.56–272.7)	0.002	-	-
Vienna All TU at LFU	15	Undef. ^a^	53.07 (5.82–483.9)	<0.001	-	-
Vienna Young TU at LFU	15	94	44.25 (4.87–401.9)	<0.001	-	-
TCGA DFS All	Undef. ^a^	Undef. ^a^	0.58 (0.14–2.47)	0.46	-	-
TCGA OS All	Undef. ^a^	52.27	1.51 (0.38–5.95)	0.56	-	-
TCGA OS Young	10.74	Undef. _a_	3.2 (0.47–21.6)	0.23	-	-
TCGA DFS Young	6.83	Undef. ^a^	16.5 (1.69–161.2)	0.02	-	-
Combined OS All	21	90.05	3.62 (1.18–11.45)	0.02	3.57 (1.53–8.56)	< 0.01
Combined DFS All	13	Undef. ^a^	2.82 (0.78–10.10)	0.11	1.68 (0.25–13.23)	0.55
Combined OS Young	10.75	Undef. ^a^	18.95 (4.03–88.87)	<0.001	-	-
Combined DFS Young	2.17	Undef. ^a^	29.65 (5.96–147.40)	<0.001	-	-

^a^ by definition Kaplan–Meier estimates of median survival cannot be computed in groups where <50% of individuals have reached the statistical endpoint (death) within the period of observation, ^b^ overexpression was defined as IHC score ≥ 4 in the Vienna cohort and as a z-score ≥1.96 in the TCGA cohort, ^c^ determined by log rank test, ^d^ determined by Cox regression analysis.

## Data Availability

All data underlying the results presented herein are included in the manuscript or are freely accessible from The Cancer Genome Atlas (TCGA) database (https://www.cancer.gov/tcga; last accessed 1 March 2021).

## Supplementary Materials

The following are available online at https://www.mdpi.com/article/10.3390/cancers13092082/s1, Figure S1: Kaplan–Meier survival curves in relation to Annexin 1 overexpression, Figure S2: Kaplan–Meier survival curves in relation to PRKCA protein overexpression in all subgroups, Figure S3: Box plots of total mutation count (log transformed) in PRKCA high versus low TCGA samples, Table S1: Clinical and proteomics data from the study cohorts. Table S2: Single nucleotide variants (SNPs) and Copy number variants (CNVs) differentially expressed in the PRKCA high versus PRKCA low samples within the TCGA cohort, Table S3: Full list of differentially expressed mRNAs, Table S4: Full list of GeneOntology hits.

## References

[1] Bray F., Ferlay J., Soerjomataram I., Siegel R.L., Torre L.A., Jemal A. (2018). Global cancer statistics 2018: GLOBOCAN. CA. Cancer J. Clin..

[2] Reshmi S.C., Saunders W.S., Kudla D.M., Ragin C.R., Gollin S.M. (2004). Chromosomal instability and marker chromosome evolution in oral squamous cell carcinoma. Genes Chromosom. Cancer.

[3] Siebers T.J.H., Bergshoeff V.E., Otte-Höller I., Kremer B., Speel E.J.M., Van Der Laak J.A.W.M., Merkx M.A.W., Slootweg P.J. (2013). Chromosome instability predicts the progression of premalignant oral lesions. Oral Oncol..

[4] Forastiere A., Koch W., Trotti A., Sidransky D. (2001). Head and neck cancer. N. Engl. J. Med..

[5] Pires F.R., Ramos A.B., de Oliveira J.B.C., Tavares A.S., de Luz P.S.R., dos Santos T.C.R.B. (2013). Oral squamous cell carcinoma: Clinicopathological features from 346 cases from a single oral pathology service during an 8-year period. J. Appl. Oral Sci..

[6] Troeltzsch M., Knösel T., Eichinger C., Probst F., Troeltzsch M., Woodlock T., Mast G., Ehrenfeld M., Otto S. (2014). Clinicopathologic features of oral squamous cell carcinoma: Do they vary in different age groups?. J. Oral Maxillofac. Surg..

[7] Sturgis E.M., Cinciripini P.M. (2007). Trends in head and neck cancer incidence in relation to smoking prevalence. Cancer.

[8] Moore S.R., Johnson N.W., Pierce A.M., Wilson D.F. (2000). The epidemiology of tongue cancer: A review of global incidence. Oral Dis..

[9] Hussein A.A., Helder M.N., de Visscher J.G., Leemans C.R., Braakhuis B.J., de Vet H.C.W., Forouzanfar T. (2017). Global incidence of oral and oropharynx cancer in patients younger than 45 years versus older patients: A systematic review. Eur. J. Cancer.

[10] Tota J.E., Anderson W.F., Coffey C., Califano J., Cozen W., Ferris R.L., St. John M., Cohen E.E.W., Chaturvedi A.K. (2017). Rising incidence of oral tongue cancer among white men and women in the United States, 1973–2012. Oral Oncol..

[11] Harris S.L., Kimple R.J., Hayes D.N., Couch M.E., Rosenman J.G. (2010). Never-smokers, never-drinkers: Unique clinical subgroup of young patients with head and neck squamous cell cancers. Head Neck.

[12] Poling J.S., Ma X.J., Bui S., Luo Y., Li R., Koch W.M., Westra W.H. (2014). Human papillomavirus (HPV) status of non-tobacco related squamous cell carcinomas of the lateral tongue. Oral Oncol..

[13] Castellsagué X., Alemany L., Quer M., Halec G., Quirós B., Tous S., Clavero O., Alòs L., Biegner T., Szafarowski T. (2016). HPV Involvement in Head and Neck Cancers: Comprehensive Assessment of Biomarkers in 3680 Patients. J. Natl. Cancer Inst..

[14] Zafereo M.E., Xu L., Dahlstrom K.R., Viamonte C.A., El-Naggar A.K., Wei Q., Li G., Sturgis E.M. (2016). Squamous cell carcinoma of the oral cavity often overexpresses p16 but is rarely driven by human papillomavirus. Oral Oncol..

[15] de Morais E.F., Mafra R.P., Gonzaga A.K.G., de Souza D.L.B., Pinto L.P., da Silveira É.J.D. (2017). Prognostic Factors of Oral Squamous Cell Carcinoma in Young Patients: A Systematic Review. J. Oral Maxillofac. Surg..

[16] Hilly O., Shkedy Y., Hod R., Soudry E., Mizrachi A., Hamzany Y., Bachar G., Shpitzer T. (2013). Carcinoma of the oral tongue in patients younger than 30 years: Comparison with patients older than 60 years. Oral Oncol..

[17] Jeon J.-H., Kim M.G., Park J.Y., Lee J.H., Kim M.J., Myoung H., Choi S.W. (2017). Analysis of the outcome of young age tongue squamous cell carcinoma. Maxillofac. Plast. Reconstr. Surg..

[18] Pickering C.R., Zhang J., Neskey D.M., Zhao M., Jasser S.A., Wang J., Ward A., Tsai C.J., Alves M.V.O., Zhou J.H. (2014). Squamous cell carcinoma of the oral tongue in young non-smokers is genomically similar to tumors in older smokers. Clin. Cancer Res..

[19] dos Santos Costa S.F., Brennan P.A., Gomez R.S., Fregnani E.R., Santos-Silva A.R., Martins M.D., de Castro-Junior G., Rahimi S., Fonseca F.P. (2018). Molecular basis of oral squamous cell carcinoma in young patients: Is it any different from older patients?. J. Oral Pathol. Med..

[20] Gu X., Coates P.J., Boldrup L., Wang L., Krejci A., Hupp T., Fahraeus R., Norberg-Spaak L., Sgaramella N., Wilms T. (2019). Copy number variation: A prognostic marker for young patients with squamous cell carcinoma of the oral tongue. J. Oral Pathol. Med..

[21] Maroun C.A., Zhu G., Fakhry C., Gourin C.G., Seiwert T.Y., Vosler P.S., Tan M., Koch W., Eisele D.W., Pardoll D.M. (2020). An Immunogenomic Investigation of Oral Cavity Squamous Cell Carcinoma in Patients Aged 45 Years and Younger. Laryngoscope.

[22] Lingen M.W., Chang K.W., McMurray S.J., Solt D.B., Kies M.S., Mittal B.B., Haines G.K., Pelzer H.J. (2000). Overexpression of p53 in squamous cell carcinoma of the tongue in young patients with no known risk factors is not associated with mutations in exons 5-9. Head Neck.

[23] Li R., Faden D.L., Fakhry C., Langelier C., Jiao Y., Wang Y., Wilkerson M.D., Pedamallu C.S., Old M., Lang J. (2015). Clinical, genomic, and metagenomic characterization of oral tongue squamous cell carcinoma in patients who do not smoke. Head Neck.

[24] Sakamoto K., Imanishi Y., Tomita T., Shimoda M., Kameyama K., Shibata K., Sakai N., Ozawa H., Shigetomi S., Fujii R. (2012). Overexpression of SIP1 and downregulation of e-cadherin predict delayed neck metastasis in stage I/II oral tongue squamous cell carcinoma after partial glossectomy. Ann. Surg. Oncol..

[25] Wang C., Liu X., Huang H., Ma H., Cai W., Hou J., Huang L., Dai Y., Yu T., Zhou X. (2012). Deregulation of Snai2 is associated with metastasis and poor prognosis in tongue squamous cell carcinoma. Int. J. Cancer.

[26] Albert S., Hourseau M., Halimi C., Serova M., Descatoire V., Barry B., Couvelard A., Riveiro M.E., Tijeras-Raballand A., De Gramont A. (2012). Prognostic value of the chemokine receptor CXCR4 and epithelial-to- mesenchymal transition in patients with squamous cell carcinoma of the mobile tongue. Oral Oncol..

[27] Liang X., Zheng M., Jiang J., Zhu G., Yang J., Tang Y. (2011). Hypoxia-inducible factor-1 alpha, in association with TWIST2 and SNIP1, is a critical prognostic factor in patients with tongue squamous cell carcinoma. Oral Oncol..

[28] Kang F.W., Gao Y., Que L., Sun J., Wang Z.L. (2013). Hypoxia-inducible factor-1α overexpression indicates poor clinical outcomes in tongue squamous cell carcinoma. Exp. Ther. Med..

[29] Almangush A., Heikkinen I., Mäkitie A.A., Coletta R.D., Läärä E., Leivo I., Salo T. (2017). Prognostic biomarkers for oral tongue squamous cell carcinoma: A systematic review and meta-analysis. Br. J. Cancer.

[30] Du L., Yang Y., Xiao X., Wang C., Zhang X., Wang L., Zhang X., Li W., Zheng G., Wang S. (2011). Sox2 nuclear expression is closely associated with poor prognosis in patients with histologically node-negative oral tongue squamous cell carcinoma. Oral Oncol..

[31] Huang C.F., Xu X.R., Wu T.F., Sun Z.J., Zhang W.F. (2014). Correlation of ALDH1, CD44, OCT4 and SOX2 in tongue squamous cell carcinoma and their association with disease progression and prognosis. J. Oral Pathol. Med..

[32] Cerami E., Gao J., Dogrusoz U., Gross B.E., Sumer S.O., Aksoy B.A., Jacobsen A., Byrne C.J., Heuer M.L., Larsson E. (2012). The cBio Cancer Genomics Portal: An open platform for exploring multidimensional cancer genomics data. Cancer Discov..

[33] Grossman R.L., Heath A.P., Ferretti V., Varmus H.E., Lowy D.R., Kibbe W.A., Staudt L.M. (2016). Toward a Shared Vision for Cancer Genomic Data. N. Engl. J. Med..

[34] Colaprico A., Silva T.C., Olsen C., Garofano L., Cava C., Garolini D., Sabedot T.S., Malta T.M., Pagnotta S.M., Castiglioni I. (2016). TCGAbiolinks: An R/Bioconductor package for integrative analysis of TCGA data. Nucleic Acids Res..

[35] Risso D., Schwartz K., Sherlock G., Dudoit S. (2011). GC-content normalization for RNA-Seq data. BMC Bioinformatics.

[36] Berenblum I. (1941). The Cocarcinogenic Action of Croton Resin. Cancer Res..

[37] Niedel J.E., Kuhn L.J., Vandenbark G.R. (1983). Phorbol diester receptor copurifies with protein kinase C. Proc. Natl. Acad. Sci. USA.

[38] Guo Y.-J., Pan W.-W., Liu S.-B., Shen Z.-F., Xu Y., Hu L.-L. (2020). ERK/MAPK signalling pathway and tumorigenesis. Exp. Ther. Med..

[39] Martini M., De Santis M.C., Braccini L., Gulluni F., Hirsch E. (2014). PI3K/AKT signaling pathway and cancer: An updated review. Ann. Med..

[40] Li W., Zhang J., Flechner L., Hyun T., Yam A., Franke T.F., Pierce J.H. (1999). Protein kinase C-α overexpression stimulates Akt activity and suppresses apoptosis induced by interleukin 3 withdrawal. Oncogene.

[41] Gupta A.K., Galoforo S.S., Berns C.M., Martinez A.A., Corry P.M., Guan K.L., Lee Y.J. (1996). Elevated levels of ERK2 in human breast carcinoma MCF-7 cells transfected with protein kinase Cα. Cell Prolif..

[42] Salama M.F., Liu M., Clarke C.J., Espaillat M.P., Haley J.D., Jin T., Wang D., Obeid L.M., Hannun Y.A. (2019). PKCα is required for Akt-mTORC1 activation in non-small cell lung carcinoma (NSCLC) with EGFR mutation. Oncogene.

[43] Wu B., Zhou H., Hu L., Mu Y., Wu Y. (2013). Involvement of PKCα activation in TF/VIIa/PAR2-induced proliferation, migration, and survival of colon cancer cell SW620. Tumor Biol..

[44] Mandil R., Ashkenazi E., Blass M., Kronfeld I., Kazimirsky G., Rosenthal G., Umansky F., Lorenzo P.S., Blumberg P.M., Brodie C. (2001). Protein kinase Cα and protein kinase Cδ play opposite roles in the proliferation and apoptosis of glioma cells. Cancer Res..

[45] Zhang H.X., Liu O.S., Deng C., He Y., Feng Y.Q., Ma J.A., Hu C.H., Tang Z.G. (2018). Genome-wide gene expression profiling of tongue squamous cell carcinoma by RNA-seq. Clin. Oral Investig..

[46] Cardesa A., Nadal A. (2011). Carcinoma of the head and neck in the HPV era. Acta Dermatovenerol. Alpina Pannonica Adriat..

[47] Dmello C., Srivastava S.S., Tiwari R., Chaudhari P.R., Sawant S., Vaidya M.M. (2019). Multifaceted role of keratins in epithelial cell differentiation and transformation. J. Biosci..

[48] Plzák J., Bouček J., Bandúrová V., Kolář M., Hradilová M., Szabo P., Lacina L., Chovanec M., Smetana K. (2019). The Head and Neck Squamous Cell Carcinoma Microenvironment as a Potential Target for Cancer Therapy. Cancers.

[49] Tampa M., Mitran M.I., Mitran C.I., Sarbu M.I., Matei C., Nicolae I., Caruntu A., Tocut S.M., Popa M.I., Caruntu C. (2018). Mediators of Inflammation—A Potential Source of Biomarkers in Oral Squamous Cell Carcinoma. J. Immunol. Res..

[50] Winck F.V., Prado Ribeiro A.C., Ramos Domingues R., Ling L.Y., Riaño-Pachón D.M., Rivera C., Brandão T.B., Gouvea A.F., Santos-Silva A.R., Coletta R.D. (2015). Insights into immune responses in oral cancer through proteomic analysis of saliva and salivary extracellular vesicles. Sci. Rep..

[51] Pfeifhofer C., Gruber T., Letschka T., Thuille N., Lutz-Nicoladoni C., Hermann-Kleiter N., Braun U., Leitges M., Baier G. (2006). Defective IgG2a/2b Class Switching in PKCα −/− Mice. J. Immunol..

[52] Lim P.S., Sutton C.R., Rao S. (2015). Protein kinase C in the immune system: From signalling to chromatin regulation. Immunology.

[53] Haabeth O.A.W., Lorvik K.B., Hammarström C., Donaldson I.M., Haraldsen G., Bogen B., Corthay A. (2011). Inflammation driven by tumour-specific Th1 cells protects against B-cell cancer. Nat. Commun..

[54] Xu W., Yang Z., Lu N. (2015). A new role for the PI3K/Akt signaling pathway in the epithelial-mesenchymal transition. Cell Adh. Migr..

[55] Lewis D.R., Check D.P., Caporaso N.E., Travis W.D., Devesa S.S. (2014). US lung cancer trends by histologic type. Cancer.

[56] Zhang L., Li M., Wu N., Chen Y., Yang F. (2015). Time trends in epidemiologic characteristics and imaging features of lung adenocarcinoma: A population study of 21,113 cases in China. PLoS ONE.

[57] Jiang H., Fu Q., Song X., Ge C., Li R., Li Z., Zeng B., Li C., Wang Y., Xue Y. (2019). HDGF and prkca upregulation is associated with a poor prognosis in patients with lung adenocarcinoma. Oncol. Lett..

[58] Isakov N. (2018). Protein kinase C (PKC) isoforms in cancer, tumor promotion and tumor suppression. Semin. Cancer Biol..

