# Supplementary material for: PRKCA Overexpression Is Frequent in Young Oral Tongue Squamous Cell Carcinoma Patients and Is Associated with Poor Prognosis
Source: Cancers (Basel). 2021 Apr 25;13(9):2082. doi: 10.3390/cancers13092082 (PMC8123332; doi:10.3390/cancers13092082)
Supplement: Supplementary file 1 [file cancers-13-02082-s001.zip › cancers-1154669-supplementary files/Supp. Figures.pdf]

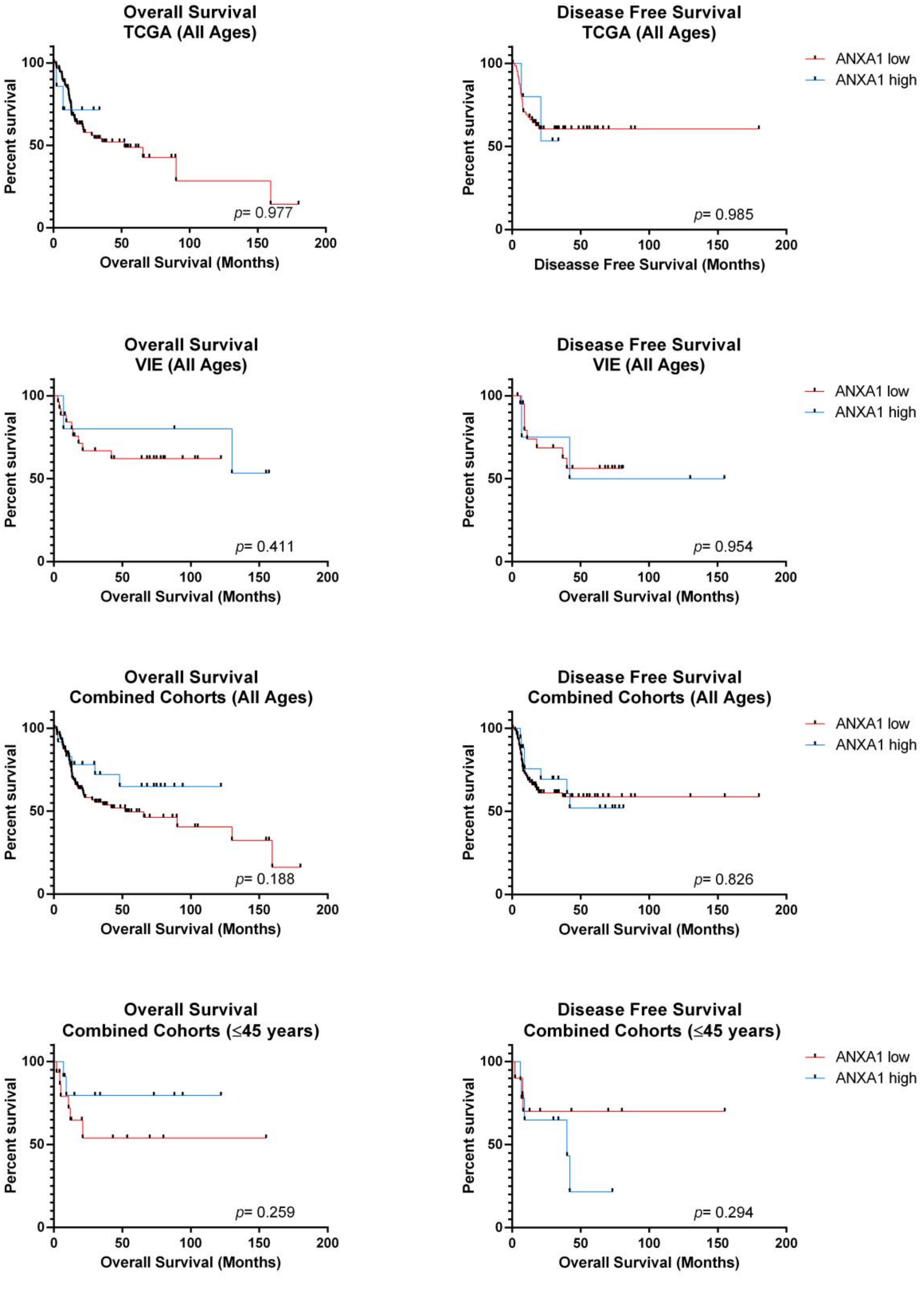

Figure S1. Kaplan–Meier survival curves in relation to Annexin 1 overexpression.

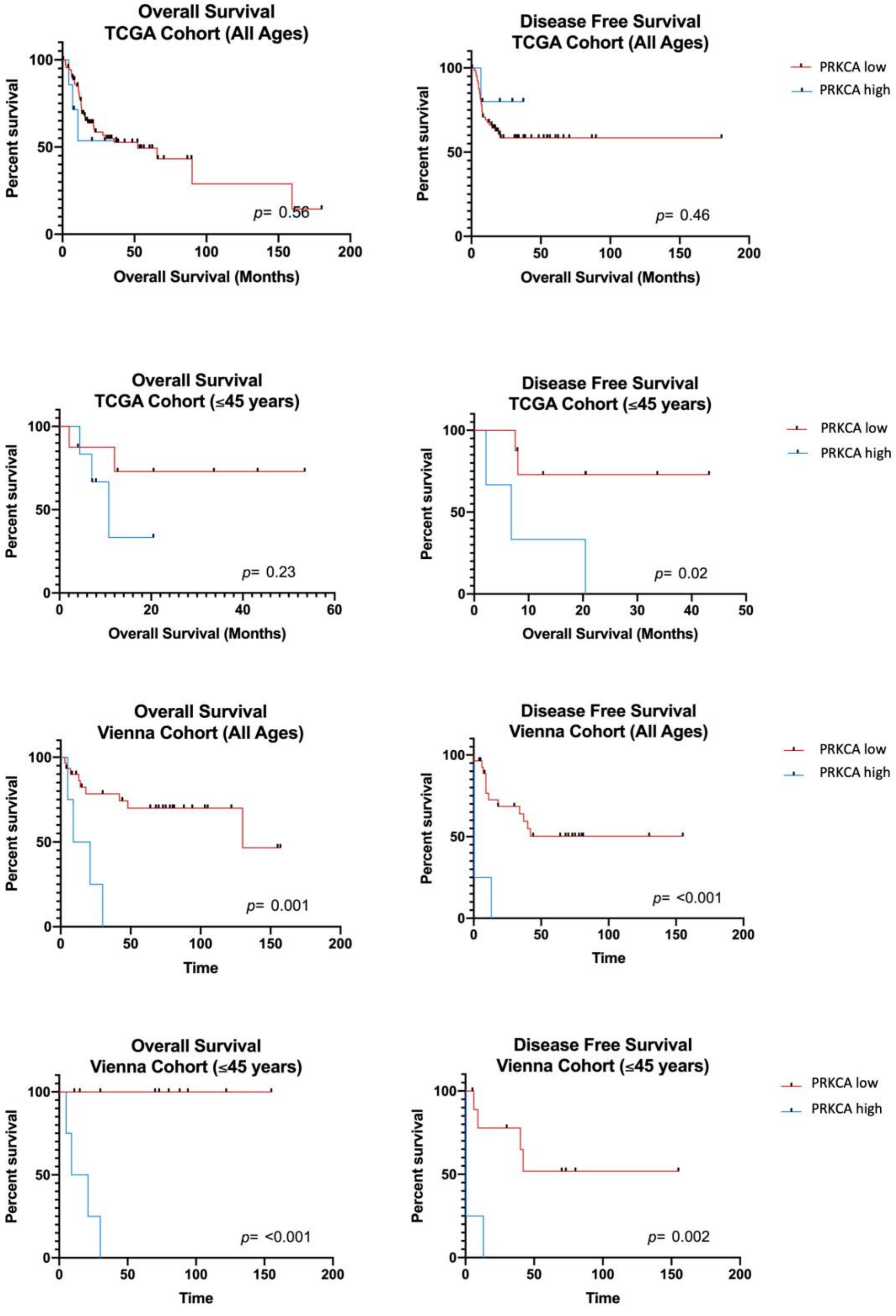

Figure S2. Kaplan–Meier survival curves in relation to PRKCA protein overexpression in all subgroups.

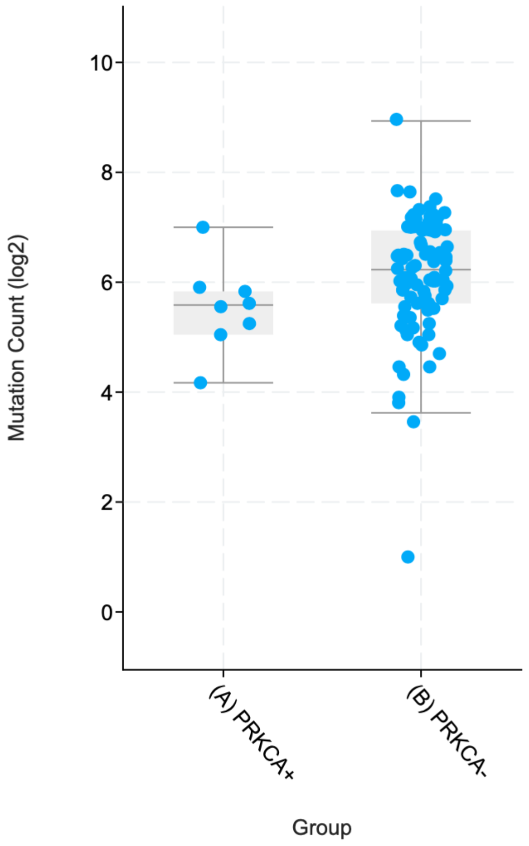

Figure S3. Box plots of total mutation count (log transformed) in PRKCA high versus low TCGA samples.
